# Representative Participation in a Large-Scale Health IT Project

**DOI:** 10.1007/s10606-022-09457-0

**Published:** 2022-12-28

**Authors:** Øivind Klungseth Zahlsen, Dag Svanæs, Yngve Dahl

**Affiliations:** 1 Department of Computer Science, Norwegian University of Science and Technology, NO-7491 Trondheim, Norway; 2 Digital Design Department, The IT University of Copenhagen, Copenhagen, Denmark

**Keywords:** Large-scale IT projects, Participatory design, Representative participation, Structural arrangements, User involvement

## Abstract

User involvement is widely recognized as best practice in the development of information technology (IT) systems. In large-scale IT projects, the involvement of users and other stakeholder groups is typically in the form of representatives, as opposed to the direct (in-person) participation characteristic for smaller projects. The potential new sharing of power that representative participation entails vis-à-vis direct stakeholder involvement, and the implications of such a shift, are an important discussion in the context of participatory design. This paper extends and adds to previous work on this subject. Drawing on stakeholder interviews conducted as part of a case study of an electronic health record implementation project in Norway, this paper seeks to describe and analyze problems that can arise with representative participation in a large-scale project. Our focus is on an observed decline of interaction between health professionals participating actively in the project and their advisory units consisting of colleagues without a formal project role. The paper describes how the project’s structural arrangements might explain this decline. The paper also describes how the participating health professionals’ involvement of the advisory units at regular intervals early in the project (broad involvement) was replaced by more ad hoc and competence-oriented approaches (narrow involvement). We further use the organizational structure of democracies as the basis for two analogies, (I) participants-as-political-representatives and (II) participants-as-technocrats. The observed decline in interaction between the participating health professionals and their advisory units can be seen as a transition in role from user representative to technocrat. Generalizing from the case, we suggest that (1) a project’s structure strongly affects the possibilities of participating users to consult other users (e.g., non-participating colleagues) about issues concerning the design solution, (2) a project’s structure conditions the role of participating users and who, or what, they represent, and (3) representative participation requires rethinking a project’s structure.

## Introduction

The involvement of users and other stakeholders is a fundamental principle in user-centered and participatory design (UCD and PD) (Norman and Draper, 1986; Kensing and Greenbaum, 2013) and is widely recognized as best practice in the development of information technology (IT) systems (ISO 9241–210, 2019). In PD, with its focus on supporting and developing the tacit knowledge and skills of the user, emphasis has been placed on users’ active participation in design processes (Ehn, 2017). Studies point out that PD projects, and particularly early initiatives, have been predominantly small scale (Clement and Van den Besselaar, 1993; Oostveen and Van den Besselaar, 2004; Simonsen and Hertzum, 2008). In smaller participatory projects, which typically target user groups of limited sizes (e.g., (Dahl and Hanssen, 2018; Lindsay et al., 2012; Malinverni et al., 2014)), we find that those who will be using the technology being designed to a greater extent also take part in PD activities. While the active involvement of *all* users is not feasible in most cases, small-scale projects typically have a higher ratio of involved users (i.e., participants) to non-involved users than what the situation is in large-scale projects. Thus, small-scale participatory projects are to a greater extent characterized by *direct* participation.

With the increasing globalization of IT and the growing size and complexity of IT projects, it is an open question as to how well the insights from earlier PD projects scale up to large-scale IT projects—In particular, how the shift from direct participation in small to medium-scale projects to representative participation in large-scale projects should be conceptualized. This is not to say that PD, historically, has disregarded issues related to representative participation (see, e.g., Grudin, 1991; Kensing and Blomberg, 1998; Bødker and Grønbæk, 1991)), but rather that the question of representativeness has become even more topical in light of the growing size of IT projects. One key challenge is that the large number of users and other stakeholders of the technology being designed in large-scale projects makes direct participation practically infeasible (Roland et al., 2017). As a consequence, involvement in larger participatory IT projects is often in the form of *representative* participation, where the stakeholders taking part in the design process are appointed or elected individuals (i.e., representatives) who are considered to act on behalf of larger stakeholder groups or organizations in matters concerning the design solution (Oostveen and Van den Besselaar, 2004; Mogensen and Wollsen, 2014; Bonsignore et al., 2016; Dalsgaard and Eriksson, 2013). Thus, a project characterized by representative participation has a lower ratio of involved users to non-involved users than projects characterized by direct participation.

The tendency to rely on user representatives in large-scale IT projects arguably makes the role of the representative highly influential with respect to the unfolding design process and its outcome. The implications of the potential transition in power that representative participation implies vis-à-vis the direct user involvement characteristic of smaller participatory projects are an important discussion in the context of participatory design. To contribute to the ongoing discourse on the challenges of scaling up PD (e.g., Pilemalm and Timpka, 2008; Hamzah and Wahid, 2016; Cozza and De Angeli, 2015; Simonsen and Hertzum, 2012; Zahlsen et al., 2021; Dalsgaard and Eriksson, 2013; Mogensen and Wollsen, 2014)), the current paper seeks to describe and analyze problems associated with representative participation as a strategy for giving users and other technology stakeholders a say in large-scale IT projects. We do so by reporting and reflecting on the case study of an ongoing health IT project intending to implement a common electronic health record (EHR) system in one of Norway’s most populated regions. The project involves more than 400 health professionals from the various medical specialties and regional healthcare services that the new EHR system is intended to serve. In project documents these professionals are referred to as *subject matter experts* (SMEs). The project documents and public description of the project, as discussed in further detail later, are ambiguous and contradictory as to the role of the SMEs—whether they are representing their regional community of practice or if they are experts providing domain knowledge to the developers?

This case study investigates aspects of the participatory work related to the development of a specific module in the new EHR system. In particular, we focus on the observed decline that occurred throughout the project’s first phases in the interaction between the SMEs and colleagues (i.e., non-participants) in advisory units in the client health organizations that were originally established to support the SMEs’ project work. Drawing on data gathered through interviews with SMEs and their colleagues, we first investigate the central features of the above-mentioned breakdown in interaction and then the SMEs’ collegial involvement practices. Using the findings from our data analysis as a starting point for reflection, we discuss how *structural arrangements* in the project—aspects about the structure and organizing of the project, including the participating stakeholder organizations—may have conditioned the SMEs’ role and collegial involvement practices, and outline the wider implications of such a perspective.

The remainder of the article is structured as follows. We continue in Sect. 2 by accounting for participation as a problem of scale and problematizing direct versus representative (indirect) participation. In Sect. 3, we present the central aspects of the project (i.e., the case) we have studied as part of our investigation of representative stakeholder participation in large-scale IT projects. Section 4 presents the methods used to collect and analyze the data for this study. The results of the analysis are presented in Sect. 5, before we discuss the key implications of the findings in Sect. 6. Section 7 accounts for key methodological considerations, and we end the paper by providing a summary of the presented work and concluding remarks in Sect. 8.

## Background

### Participation as a problem of scale

*Participation*, as understood in PD, is a notion with strong political connotations. In comparison with, for example, UCD, where the involvement of users is primarily considered a means to ensure usable design solutions (Norman and Draper, 1986), user participation in PD has a strong emancipatory agenda: Participation involves a *sharing of power* between those using and affected by technology, on the one hand, and those developing or owning the technology, on the other (Bratteteig and Wagner, 2014; 2012; Bjerknes and Bratteteig, 1995; Clement and Van den Besselaar, 1993; Robertson et al., 2006; Robertson and Wagner, 2012; Beck, 2002; Floyd et al., 1989). Hence, a fundamental premise in PD is that people should have a *right* to be included in the design of technology that will affect their everyday lives.

PD’s emancipatory agenda is particularly reflected in what Kensing and Greenbaum (2013) considered to be enduring guiding principles of PD. For example, *equalizing power relations*, *situated-based actions*, *mutual learning,* and *tools and techniques.*

Common to the guiding principles of PD mentioned above is that they tend to rely on the establishment of trust, close interactions, and empathic relations between designers and users. The importance of developing quality designer–user relations is also reflected in PD practice, such as in the seminal UTOPIA project (Bødker et al., 1987) and in contemporary studies (e.g., Dahl and Svanæs, 2020; Lindsay et al., 2012; Light and Akama, 2012). Roland et al. (2017) used the term *singular PD* to describe the direct one-to-one relation between developers and situated users that custom PD techniques such as prototypes, mock-ups, and workshops typically imply. In PD, these techniques have been considered to play a central role in activating tacit knowledge of participants and evoke their critical reflection when it comes to design solutions (Bossen, 2006).

As PD methods and techniques are increasingly applied in large IT projects, many of the presumptions about the participatory context that underlie singular PD, are no longer valid. For example, Pilemalm and Timpka (2008) described scale-related participatory challenges, such as involving entire user groups in design-related activities, heterogeneity in user groups, and user group discontinuity. Dalsgaard and Eriksson (2013) highlighted intergroup communication as one of the challenges that large participatory projects involving several stakeholder groups can bring about. Braa and Sahay (2013) discussed PD in the context of developing countries and in a longitudinal perspective, which involved taking into further consideration technological and political changes over time and related challenges.

To remain a vital design approach, Bødker and Kyng (2018) argued that PD *needs* to face “big issues” such as scaling. However, challenges associated with Third generation PD, in many ways question the extent to which the PD methodology can be applied or adapted to large IT projects without significantly compromising its emancipatory agenda.

### Directness of participation

Historically, the upscaling of participation for democratic purposes is by no means a new challenge. In the development of most modern-day Western democracies, the use of representatives—that is, persons who are elected by citizens to represent their interests in the governance of a country—can be seen as a measure invented to deal with the issue of participatory scaling (Dahl, 2008, pp. 213–224) and the impracticalities and costs often associated with direct democracy (Przeworski, 2010, p. 110). The extent to which representative democracy can be considered truly democratic is debatable, as reflected in political science literature. For example, drawing on Dahl’s (2008, pp. 108–114) democratic criteria, Landemore (2017) argues that representative democracy has turned into an “exclusionary paradigm,” which fails to meet key standards such as effective participation, enlightened understanding, and control of the agenda.

In spite of its potential weaknesses when it comes to empowering citizens, the model of representative democracy has been adopted in contexts far beyond democratic state governance. Variants of the model can be found in large parts of democratic societies, including, for example, municipal councils (Fung, 2003; Michels and De Graaf, 2010), student councils (Flint and O’Hara, 2013) and work environments (Abildgaard et al., 2020).

Representative participation has also been adopted as a mode of user involvement in large IT projects, where the user population of the solution being developed is generally too large for direct participation to be feasible. For example, in the Danish project *Fablab@school.dk* (Bødker et al., 2017), which was established in the wake of an educational reform emphasizing the use of digital technology in schools, representatives from different levels of authority were engaged. This ranged from representatives from parliament, national agencies, and municipalities down to school committees and management, teachers, and students. Stakeholder representatives also play a central role in the *Fasme* project (Oostveen and Van den Besselaar, 2004) which aimed at facilitating EU citizens’ mobility by streamlining time-consuming bureaucratic obstacles. Eight classes of users at different organizational levels, each class represented through a set of representatives, were involved in the design of a prototype E-government system.

Representative participation in IT projects, however, has been problematized in PD-related literature. For example, it has been argued that successful solution development depends not only on user representatives being present and how much, but to a large extent *who* the user representatives (i.e., the participants) are. Markus and Mao (2004) proposed that having representatives from a larger portion of stakeholder groups (e.g., operational users, their management personnel, and relevant external stakeholders) is beneficial in this regard. An interview study of project managers’ strategies when it comes to selecting users for participation in an IT project, however, revealed that the managers favored individuals that had the ability of advocating a vision for the system (i.e., *user advocates*) and championing its implementation in the organization (i.e., *system champions*), over persons who were representative of the full range of users (Rasmussen et al., 2011). The establishment of a core group of participants capable of not only reflecting on their practice, but also of communicating and pioneering PD initiatives within the stakeholder organization, was also described as a central participant selection strategy in a study by Iversen and Dindler (2014).

In the light of PD’s emancipatory agenda, representative (indirect) participation is, in many ways, controversial. PD has traditionally focused on the direct participation of users and other stakeholders as a means to understand *knowledge by doing* (Schön, 1983; Hayes, 2014; Muller et al., 1993): the tacit knowledge and “invisible work” (Nardi and Engeström, 1999) that often go into performing everyday activities, and how technology may support, rather than disrupt, experienced-based ways of problem solving. Early CSCW research on groupware (Pipek and Wulf, 1999) also revealed the benefits of directly involving all potential users in various design issues, including choosing and reorganizing the work process to support, configuring, and developing associated functionality reliably, and in sustaining a high level of interest in the ongoing change process.

As such, direct participation has been seen as an important means to provide users genuine influence in design. Given how PD has traditionally conceptualized knowledge as inseparable from doing (i.e., practice) (Kensing and Greenbaum, 2013), representative user participation is, in theory, a compromising factor when it comes to delegating power to the users – particularly in cases where the user representative is representing heterogenous user groups.

### Positioning the current study

We consider the theoretical challenge associated with representative participation, as explained above, to be a strong motivation for conducting an empirical investigation into participants’ role and impact in a large-scale IT project. Our intention is not to verify or reject whether representative participation leads to disempowerment, but rather to offer a qualitative understanding of aspects that can affect the stakeholder representative’s role and role-associated practices. As such, the current study is to a large extent influenced by Bratteteig and Wagner’s (2016) multi-dimensional understanding of the concept of participation. In particular, we address the “What shapes participation?” dimension of Bratteteig and Wagner’s conceptualization. Accordingly, we attempt to see the project-related interactions (and breakdown in interactions) between the stakeholder representatives in the project and their non-project affiliated colleagues from the perspective of structural arrangements (pertaining to the project and the organizations involved in the project), which, according to Croziers (1973), “condition the rules of the game” and thus express “the logic” of institutions or structures.

## Case study: regional EHR implementation project

The study presented in this article was conducted in the context of an ongoing large-scale EHR system development project. Our understanding of the project is based on our interpretation of publicly available documents. In addition, this part of the paper has been fact checked with the project management.

The project’s aim is to implement a common EHR system for all primary and secondary healthcare services in Central Norway and is the first instantiation of the national strategy *One Citizen – One Health Record* (Det kongelige Helse- og omsorgsdepartementet, 2012). The EHR system is intended to support a region with a population of 720,000 and with 40,000 healthcare professionals. The estimated cost of the project is EUR 270 million.

The project is an iterative development project with a strong focus on the involvement of various stakeholder representatives from medical specialty areas and health care services in all project phases. Despite such a focus, *user empowerment* and *workplace democracy*, as understood in the PD tradition (Spinuzzi, 2005; Simonsen and Robertson, 2012), is not a specified project goal. As a procurement-through-tender project, the signed contract works as the overall requirement for the project and regulates the legal relationships between the stakeholder organizations, the budget, and the overall time constraints. The project’s ambition to give a large number of health professionals a say in the implementation of the EHR system nevertheless makes the project an interesting case to study from a PD perspective. Studies of large-scale projects that rely heavily on user participation are relatively scarce. In many ways, this scarcity makes projects, such as the one addressed in this study, unique cases from which PD can learn and potentially evolve, especially when it comes to the challenges of upscaling user participation. Dalsgaard and Eriksson’s (2013) investigation of large-scale participation in the development of The Urban Mediaspace Aarhus is one example of how PD as a field can be informed by case studies of project that are not participatory in a traditional sense, due to size, decision structure, political context, etc., Braa and Sahay’s (2013) work in context of the *Health Information Systems Programme* (HISP) initiative is another example.

In the following sections, we present some central *structural arrangements* (Bratteteig and Wagner, 2012) pertaining to the project. This includes the involved stakeholder organizations, the project’s decision structure, and the project timeline and phases. We also describe the stakeholder representatives and their intended roles, and account for the advisory units that were established in the client organizations to support the representatives taking part in the project.

The EHR project presented here was also investigated as part of a recent study by Ellingsen et al. (2022) who investigated tensions between national and local concerns in preparations of the EHR system. As such, Ellingsen et al. (ibid.) focus on other aspects of cooperation and on other stakeholders (i.e., health program managers and general practitioners) than we do in the current study.

### Project stakeholder organizations

In 2012, the program responsible for the procurement of the EHR system was initiated. A US software company was contracted as the EHR system vendor in March 2019 after a tendering process. The following month, the regional health authority and the region’s largest municipality (i.e., the *client organizations*) established a joint-stock company (i.e., the *project organization*). The main purpose of the newly formed project organization was to manage the contract and to make sure the new EHR system solution is developed according to needs and requirements of the regional health authority. This also involved ensuring that the system installations could be customized to meet the specific needs of the hospitals, municipal healthcare services, and primary care physician offices in the region.

At the time of the study, the project consisted of five stakeholder organizations:

- Client organization 1: The regional public health authority.
- Client organization 2: The municipality of the region’s administrative center.
- Client group: Primary care physician offices organized as independent business owners.
- Project organization: A joint-stock company owned by the client organizations.
- Vendor organization: The EHR system vendor, consisting of multiple divisions.

The regional public health authority (Client organization 1) runs and coordinates the region’s state-owned hospitals. When it comes to IT infrastructure, each individual hospital does not have the same autonomy as in other matters, as they are obliged to use the regional EHR system. Managing the transition from the current EHR systems to a single EHR system that can be used by all clinicians in the region and that supports standardized and efficient workflows is therefore the main interest of the regional public health authority.

The municipality (Client organization 2) currently has its own EHR system, different from the current system in the hospitals. This EHR system exists in various configurations, which makes local installments incompatible with each other. The overall aim of the project from the perspective of the client organizations and the client group is implement a new EHR system that can allow for more coherent healthcare services for patients and health professionals in region (Helseplattformen, 2019).

The project organization is a company that first and foremost aims at a successful implementation of the new EHR system, thereby having a patient administrative system ready for the hospitals and participating municipalities in the region by the spring of 2022. The company has involved more than 400 health professionals (SMEs) from the two client organizations and the client group. As previously noted, the official description of the project (in Norwegian) is ambiguous as to the role of SMEs. In some parts of the project description (Helseplattformen, 2019), the SMEs are explicitly described as representatives of their regional community of practice^1^, while in other parts (Helseplattformen, 2020) they were described as experts in a medical specialty^2^. These two descriptions contradict each other in terms of who the SMEs represent in the project (i.e., non-participating colleagues or medical expertise), and further motivate an investigation into the actual role the SMEs have played in the project.

In addition to the SMEs, the company has engaged more than 200 *application analysts*, health professionals from the client organizations who are trained by the vendor to configure the EHR system in accordance with the design decisions of the SMEs. The application analysts have a similar role as the *physician builders* described in Bansler’s (2021) study, in the sense that their responsibility is to help optimize the configuration of the system. However, the application analysts are employed for the duration of the project, while the physician builders Bansler (ibid.) draws attention to work in the post-implementation phase.

**Figure 1.**
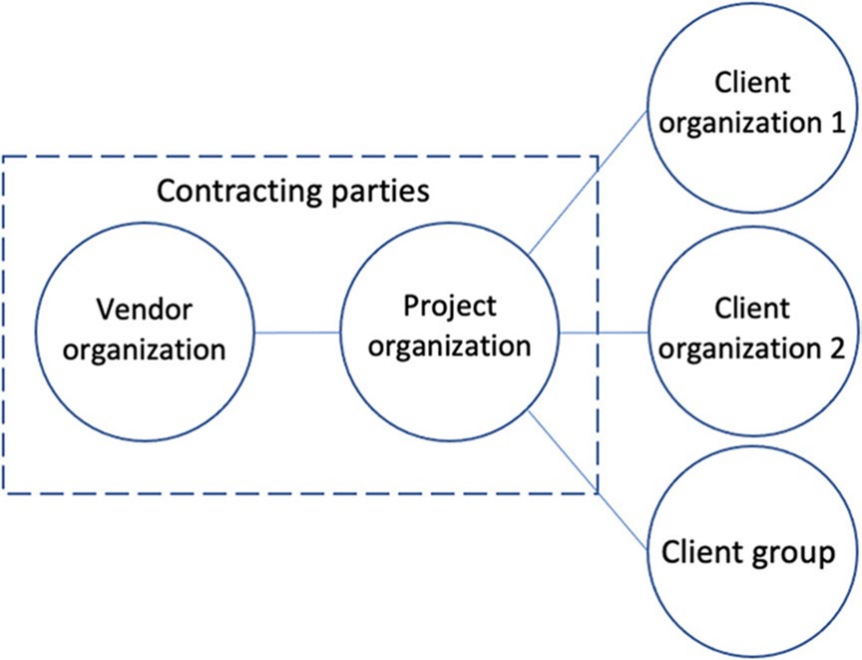
Conceptual diagram of the EHR implementation project and the interaction between the five involved stakeholder organizations.

The EHR system vendor is headquartered in the US and has relocated 30 employees to Norway for the duration of the project. The vendor’s product is a configurable EHR system. This system is delivered as several tailor-made installations, both in the US and worldwide. The vendor’s main interest is to fulfill the contractual commitments. In addition, it has a strategic interest in securing a regional foothold in the national health care system. Figure 1 presents a conceptual diagram of the interactions between the five stakeholder organizations.

### Decision structure

The project organization has established a four-level (levels 1–4) decision structure to handle emerging issues related to the configuration of the new EHR solution (Helseplattformen, 2020). Decisions on a given level are to be based on agreement. If a consensus concerning an issue cannot be achieved at that level, the issue is moved up to the next level in the decision structure.

At Level 1, the lowest level of the decision structure, are the SME groups that make decisions regarding the configuration of specific EHR system modules. These SME groups are also responsible for verifying that the configuration (performed by application analysts) is in accordance with the decisions made.

Level 2 of the decision structure consists of a decision group with members (*subject coordinators*) from all client organizations. This group also makes decisions on issues of a more principal and strategic character.

Level 3 of the decision structure consists of the client organizations’ internal steering committees, while Level 4 (the top level) consists of two members of each of the two client organizations who will make decisions on questions concerning the scope of the contracts, possible expansion of the collaboration, and other issues at top management level.

### Project timeline and phases

**Figure 2.**
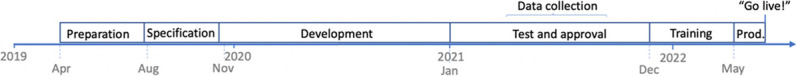
Project timeline and phases.

The project organization has developed an implementation plan for the EHR system consisting of six phases leading up to the deployment of the final solution (“Go live”): (1) preparation phase, (2) specification phase, (3) development phase, (4) test and approval phase, (5) training phase, and (6) production setting. Figure 2 illustrates the project timeline and when the data for the current study was collected relative to the timeline.

During the preparation phase, the project organization was established, detailed project plans were made, and a mapping of existing systems and work routines was carried out. In addition, the 400 SMEs were included in this phase. During the specification phase, the SMEs worked primarily with meeting facilitators from the project and vendor organizations and with developers from the vendor organization. The work consisted mainly of preparing workflows while aiming for a preliminary version of the EHR system. In the development phase, workflows were further developed, and the preparation of a common medical terminology commenced. For the test and approval phase, a large number of test users will take part in the testing of workflows. Integration between system modules is also part of this phase. The training phase focuses mainly on training health care personnel in the use of the new EHR system. The production phase focuses on deploying the system in the client organizations.

### Subject matter experts (SMEs)

The project is characterized by a large degree of involvement from health professionals (i.e., SMEs) throughout all phases of the project. After the project organization requested the participation of SMEs as part of the project’s preparation phase, health professionals employed in the client organizations and the client group were appointed to the role either directly by their superiors or through an application process. Client organization 1 (the hospitals) selected SMEs to cover most medical specialties in the region. Client organization 2 (the municipality) selected SMEs to cover the most relevant healthcare services affected by the new system. The SMEs are hired by the project organization to participate in the project on a full-time (100%) or part-time (20%, 40%, or 60%) basis. After the project, the SMEs will also likely become users of the implemented EHR system as part of their professional work.

In the EHR project, the SMEs work closely with application analysts from the project organization and developers from the vendor in configuring the EHR system or adding new functionality when required. The SMEs are typically organized in groups, each managed by a leading SME (full-time position) and are responsible for the configuration of modules relevant to the medical specialty or health care service each SME represents. The SME groups are typically given weekly configuration tasks by the EHR system vendor. Most SME groups are temporary and discontinued when their assignments have been completed, while some groups are more permanent and meet over a longer period of the project’s timeline.

The leading SMEs are responsible for taking issues emerging as part of the configuration work into regional consensus groups for further discussion and anchoring in the professional communities (see below). All leading SMEs are part of a common group at Level 1 in the decision-making structure.

### Consensus groups

To support the SMEs and help anchor decisions related to the configuration of the new EHR system in the professional communities, the regional public health authority (client organization 1) decided to establish 12 consensus groups during the spring of 2019. The consensus groups were established as advisory units within the client organizations and were consequently not part of the project organization’s formal decision structure, described in Sect. 3.3. At the time of establishment, the consensus groups consisted of 16–25 mainly non-project-affiliated health professionals employed in the client organizations. Members of the consensus group were appointed by managers in the client organizations.

**Figure 3.**
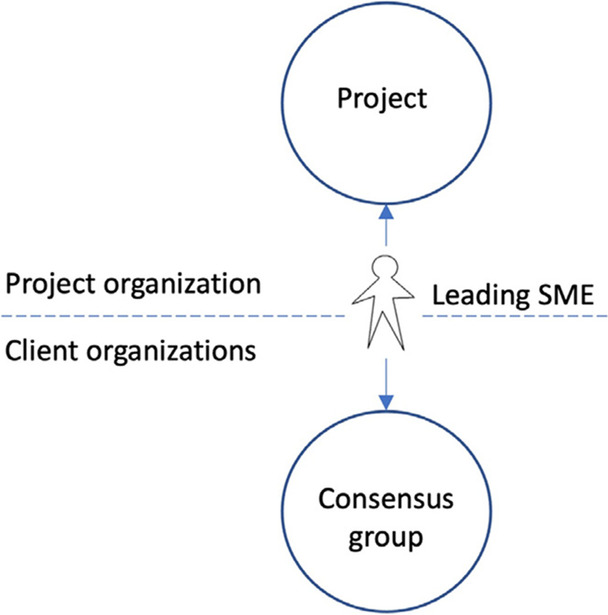
The leading SMEs’mediating role between the project and the consensus groups.

The project management decided to let the respective leading SMEs lead the consensus group meetings. The leading SMEs were thus given a new role in the project in addition to being medical experts – in these meetings they became the “interface” between the project and the future end-users of the system (Figure 3). The project documents available to us do not make explicit whether the leading SMEs were asked to see themselves as representing the project or as representing their colleagues in these meetings.

The establishment of the consensus groups and the leading SMEs as interfaces between the respective groups and the project further highlight the potential ambiguity associated with the leading SME role. On the one hand, the SMEs were invited to participate in the project based on their knowledge and experience in a medical specialty. On the other hand, being asked to lead the consensus group meetings also potentially gave them a representative role as the consensus groups were invented to help anchor decisions affecting the implementation of the EHR system in the client organizations. The latter suggests that the leading SMEs were not intended by the project management to be sole decision-makers in light of their medical expertise, but rather also to some extent to be representatives of a community of practice.

## Methods

### Respondents

To develop a comprehensive understanding of the issues related to representative participation in the EHR implementation project, we conducted a qualitative study consisting of semi-structured interviews with SMEs and their non-project-affiliated colleagues in the client organizations. Most of the interviewed SMEs were involved in the development of a specific EHR system module. As such, the interviewed SMEs do not represent a cross-section of SMEs involved in the project but are a rather a collection of participants collaborating on a particular part of the new EHR system. Several of the interviewed SMEs had developed professional relations with one another prior to the project.

A sample of 14 respondents (R1–R14) was recruited for the study. This included five leading SMEs, two SMEs, and seven colleagues, six of whom had acted as consensus group members. The age of the respondents ranged from 28 to 67 years (M = 45; SD = 10.22). Four respondents described themselves as male, while ten described themselves as female.

The respondents were recruited using a combined strategy of *purposive sampling* and *snowballing sampling* (Oates, 2006, p. 98). Most of the leading SMEs and SMEs that were interviewed had taken part in project meetings we had previously observed as part of earlier research activities in the project (Zahlsen et al., 2020; Zahlsen et al., 2021). By purposely recruiting respondents from observed project meetings, we hoped to benefit from a contextual understanding of the project and its activities when interpreting the collected data. Non-project affiliated colleagues of the SME were recruited based on the recommendation of interviewed SMEs (i.e., “snowballing”) and were typically individuals that a specific leading SME reported to frequently consult on project-related matters.

### Interview protocol

To guide the interviews, a protocol containing questions related to the topic of representative participation was developed. The interview questions in the protocol were inspired in part by observations we have made in previous research projects within the same program (Zahlsen et al., 2020, 2021) and addressed the following topics: *the role of the representative*, *collegial involvement practices*, and *perceived influence on the project*.

### Data collection and analysis

The interviews were conducted over a period of seven months, from April to October 2021. The data collection period commenced in the early stages of the project’s test and approval phase (Figure 2) after the preparation, specification, and development phases had been concluded. This potentially gave the respondents the opportunity to reflect on their experiences from two entire project phases (most of the respondents were hired as SMEs by the project organization during the preparation phase). The relatively long data collection period was not initially planned for, but a consequence of logistical problems associated with Covid-19 pandemic at the time of the study and the related challenge of finding suitable time slots for conducting interviews.

While all the topics in the interview guide were addressed in each interview, the questions pertaining to the different areas were asked only in cases where further reflection on a given topic was warranted. Each interview lasted between 45 and 60 min and was conducted one-on-one using Zoom video-telephony (mainly due to the COVID-19 situation at the time of the study). All interviews were conducted by the first author and digitally recorded. The audio recordings from the interviews were auto-transcribed using Microsoft Word 365.

The transcriptions were initially coded using a stepwise-deductive inductive method (Tjora, 2019, pp. 3–8). The method implies following an inductive (bottom-up) coding process where concepts are developed from thematically coded empirical material, and where deductive (top-down) loops act as formal quality checks at each step of the analysis.

The first step in the coding process was conducted by the first author and involved listening to the audio recordings multiple times, while also looking through the auto-transcriptions in search of passages or excerpts pertaining to the topic of representative participation. Relevant excerpts in the transcripts were marked and manually checked against the audio recordings to ensure that the excerpt had been correctly transcribed before being given a code using words or phrases from the excerpt to ensure empirically close codes.

The second step of the coding process involved assembling the codes into higher-order code groups labeled with a descriptive text. For each code group, the related original excerpts in the transcripts were cross-checked to verify that a suitable code group label—that is, one that reflected the wording in the empirical material—had been used. All authors took part in this step.

In the third and final step, the code groups were discussed in meetings between the authors and grouped into concepts. The discussions also led to the refinement of some of the code groups. The concepts of relevance for the topic of this paper (i.e., the role and practices of stakeholder representatives) are presented in Sect. 5.

**Figure 4.**
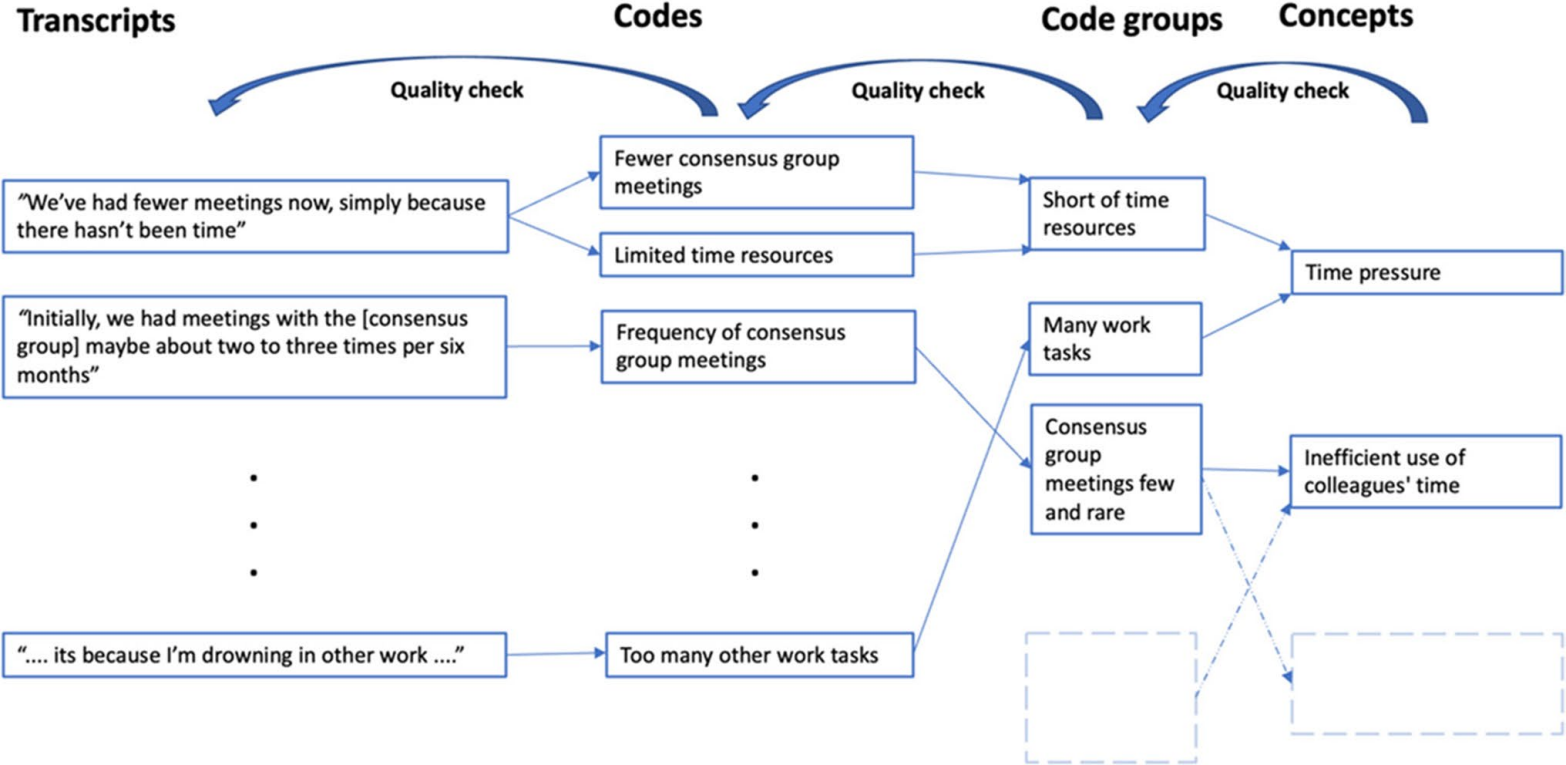
Illustration of the coding process.

Figure 4 exemplifies the general coding process by showing how some of the excerpts from the interview transcripts formed the basis for codes, which then lead to code groups and eventually concepts.

## Results

The interviews offered a rich set of data on the respondents’ perceptions of their own roles, role-related practices, and issues related to participation in the project. The results presented below, however, are limited to a particular development in the project—that is, a decline in the interaction between the leading SMEs and the consensus groups. This focus did not come as a result of an a priori intention to investigate the “breakdown” in interaction as a phenomenon. Rather, it is a consequence of our inductive coding approach to the interview data and how we, through the approach, found the declining influence of the consensus groups to be a recurring issue in the respondents’ answers to the interview questions. As specified later, the coding process also helped us become aware of both features that may explain the breakdown and how collegial involvement practices of the leading SME changed as a result.

Specifically, the analysis helped reveal five concepts, all pertaining to the declining interaction between the leading SMEs and the consensus groups throughout the project’s first phases. The first three concepts pertain to features of this breakdown:

1. Time pressure
2. Inefficient use of colleagues’ time
3. Communication and understanding
  The last two concepts describe the leading SMEs’ collegial involvement practices after the breakdown:
4. “On-demand” involvement
5. Discussion partners

In the following subsections, we first explain the breakdown in further detail before an elaboration of each of the five concepts noted above is provided. As part of accounting for the concepts, we present quotes from the interviews that we consider central to the respective concepts, along with our interpretations of the quotes. To make the individual quotes referable in the text, each quote is marked with a unique identifier along with a reference to the quoted respondent and their project-related role (e.g., “Quote 1, R8, leading SME”).

### The “Breakdown”

As stated earlier, we direct particular attention in this paper toward a phenomenon pertaining to the case study, that is, the breakdown that occurred in the project regarding the interaction between the leading SMEs and the consensus groups. As the project progressed toward the specification phase, the project organization expected the leading SMEs to manage and organize meetings with the consensus groups. However, at a relatively early stage in the development phase, the interest among the leading SMEs in meeting with the consensus groups to discuss project matters appeared to be declining. The group of leading SMEs (at Level 1 in the decision-making structure) requested that the consensus group meetings no longer be considered a mandatory activity, which the subject coordinators (at Level 2 in the decision structure) approved. Because of this breakdown, the consensus groups lost their potential influence on project decisions and were replaced by other means of collegial involvement, as described later in the paper.

### The declining influence of the consensus groups

#### Time pressure

One central aspect that may explain the breakdown relates to a perceived mismatch between the continuous demands of the project and the extent to which the consensus groups were able to help the leading SME fulfill their project responsibilities in a timely and efficient manner. One leading SME explained:

Initially, we had meetings with the [consensus group] maybe about two to three times every six months. That’s the group with members who are not [SMEs], but who are meant to support them [the SMEs]. We’ve had fewer meetings now, simply because there hasn’t been time—and because I’ve chosen to send questions directly to individuals in the group to avoid having to bring them up in large meetings, but instead get the concrete answer without needing to arrange a meeting where I’m supposed to deliver something... It didn’t really make sense to arrange meetings and constantly present something, when there are issues that we need prompt feedback on... In this phase [development phase], where we build content, I can’t wait two months, until the next meeting, to get an answer*.* (Quote 1, Respondent 8, leading SME)

This statement describes two challenges, both related to temporal aspects of the leading SME’s interaction with the consensus group. The first challenge relates to the amount of time required for preparing and facilitating meetings with the consensus group, vis-à-vis the time available for the leading SME to fulfill project-related work tasks and deadlines (“It didn’t really make sense to arrange meetings and constantly present something, when there are issues that we need prompt feedback on*.*”). The perceived limited return of time invested in meetings with the consensus group caused the leading SME to reduce the frequency of the meetings (“We’ve had fewer meetings now, simply because there hasn’t been time*.*”) and also to change the form of interaction with the consensus group (as further elaborated in 5.3).

Second, the leading SME points out the problem of asynchrony between consensus group meetings and project deliverables—Too infrequent meetings will not provide timely answers that the leading SME needs in order to fulfill responsibilities within the project and meet the deadlines set for deliverables (“I can’t wait two months, until the next meeting, to get an answer”).

Based on the above, the *time pressure* the project has put on leading SMEs can be seen as a central reason for the breakdown. The gradual decline in the interaction between the leading SMEs and the consensus groups likely escalated the consensus groups’ challenges of keeping up with the pace of the project. This situation reduced the possibilities for the consensus groups to help the leading SMEs solve pressing issues in a timely manner.

#### Inefficient use of colleagues’ time

Another aspect that may explain the breakdown relates to the spending of time resources in the client organization in relation to the project and, in particular, the consensus group meetings:

And then we saw that what we got in return [from the consensus group] was perhaps not what we really needed—that maybe we instead needed use other forums, or e-mail, or address individuals directly—because the [consensus group] is so broadly composed that it doesn’t make sense, for example, for an assistive technology technician to sit and listen to things that don't concern him, but that I should instead arrange a meeting with the assistive technology technicians about issues that concern them, and use e-mail for minor issues for things to go quickly. (Quote 2, R8, leading SME)

From the perspective of the leading SME, project-related issues that require the attention of colleagues in the client organizations typically concern only individuals with specific work responsibilities or professional competencies (“it doesn’t make sense, for example, for an assistive technology technician to sit and listen to things that don't concern him”). Rather than opting for broad involvement of colleagues in plenary discussions of issues, the leading SME considers it more efficient, potentially for all parties, to consult those that the issues concern directly (“but that I should instead arrange a meeting with the assistive technology technicians about issues that concern them”). The respondent also appears to select the forum in which to raise issues based on whom and how many the issues concern (“use other forums, or e-mail, or address individuals directly”) in addition to efficiency considerations (“for things to go quickly”).

A key feature of the breakdown, then, appears to be linked not only to personally perceived time pressure (cf. 5.2.1), but also to the respondent’s concern over the inefficient use of colleagues’ time the consensus group meetings represented.

As the following quote suggests, leading SMEs also considered it unnecessary to consult project-external resources (e.g., the consensus groups) in cases where they perceived the required competencies to already be present internally (i.e., in the project organization):

The need to use the [consensus group] is somewhat reduced. And because we—especially when it comes to the home service, at least—have very broad competence among the [SMEs], we don’t really have a great need for those meetings. (Quote 3, R5, leading SME)

While Respondent 5 does not explicitly state that efficient use of colleagues’ (or personal) time is the motivation for relying less on the consensus group (“The need to use the [consensus group] is somewhat reduced*.*”), we consider this to be a plausible interpretation of the statement.

#### Communication and understanding

The third feature of the breakdown the analysis revealed relates to challenges in the communication and understanding between the leading SMEs and the consensus groups they interacted with. One leading SME expressed the following regarding her experiences communicating with the consensus group:

When you work on a project like this, conveying information to others who are not working directly within the project can be a bit challenging, because it’s characterized by almost like a tribal language. We speak, like, [the vendor name]-ish now, with [the vendor name] words and expressions, which not all others understand. So, like if I say [project-internal expression for specific EHR system module], then everyone in the [project organization] understands what I'm talking about, but if I say that to someone who’s not working in the [project organization], he will not know what [the expression] is…So, when you convey information to others, you have to make it as simple [for them] as possible. (Quote 4, R3, 26.00, leading SME)

According to the respondent quoted above, project affiliates have, over time, developed an internal terminology (“tribal language”) to describe various aspects of the EHR system being implemented. While there is a mutual understanding of the terminology within the project, allowing for efficient communication, the respondent expresses that the same terminology would not have meaning for anyone outside the project (“We speak, like, [the name of the EHR system vendor]-ish now, with [the name of the EHR system vendor] words and expressions, which not all others understand*.*”). To reduce confusion, the respondent expresses being mindful of not using project-internal terminology when communicating about the new EHR solution with non-project-affiliated colleagues, instead using more generic terms they are more likely to comprehend. The “language” barrier indicated by Respondent 3 can be regarded as impeding efficient communication and degree of understanding between leading SMEs and consensus groups.

Throughout the analysis, we also became aware of other challenges related to communication and understanding between those inside and outside of the project. For example, one of the SMEs explained how he found it problematic to demonstrate project-related work in progress to colleagues who had not been involved in the development process.

I think it has been difficult to demonstrate a half-completed solution… Before this was completed, I was thinking: "Ouch! This isn’t complete and there’s something wrong with the [terminology]", and I was a little worried that there would be resistance [among colleagues]. I think one should wait to demonstrate [the EHR system] until one has a solution that is as complete as possible. One could, of course, argue that one is supposed to sow motivation by demonstrating parts [of the EHR system]—invite some colleagues into my office, who can say: «Oh, that's what it looks like». But to start demonstrating too much before I know the solution fine myself—and before it is finished—I find that a bit difficult, because it quickly becomes a bit like: "Even he doesn’t get it!" That doesn't necessarily serve any purpose. (Quote 5, R1, SME)

Respondent 1 expresses reluctance toward showing colleagues solutions that are currently being developed, due to a concern that aspects that are incomplete (e.g., terminology in the user interface) may cause misunderstandings and thus lower colleagues’ acceptance of the new EHR solution. To avoid such a risk, Respondent 1 expresses a need to understand how to use the new EHR solution personally before demonstrating it to others. The interviews also revealed that other SMEs held similar attitudes.

As expressed by one consensus group member, the reluctance among several SMEs and leading SMEs toward demonstrating preliminary results made it challenging for project externals to understand, comment on, and contribute to inform the development work:

When [the leading SME] say "this,” what do they mean by "this"? We are struggling with communicating. For example, when we talk about [patient] records, we, who are not [SMEs], envision [the existing EHR system] in our minds…while [the leading SME], when she asks [a design-related question], is envisioning the entire [new EHR system]. So, there is something about defining the terms being used: what do they mean? (Quote 6, R10, consensus group member)

Respondent 10 emphasizes that, in the lack of demonstrations of work in progress, her—and other consensus group members’—main reference point is the EHR system currently in use in the client organizations (which is to be replaced). As a consequence, consensus group members’ and the SMEs’ mental models of the new EHR system are likely to differ considerably.

Based on the above, we consider the lack of both a shared terminology and concrete design artifacts from the development work to help facilitate communication and understanding between “insiders” and “outsiders” as central features of the breakdown.

### Collegial involvement practices after the breakdown

Having described key findings related to the declining influence of the consensus groups, we now turn our attention to how the project-related collegial involvement practices of the leading SMEs continued *after* the breakdown.

#### “On-demand” involvement

One recurring pattern in the way leading SMEs described their post-breakdown practices of collegial involvement was characterized by a more ad hoc, competence-oriented approach. The following quote gives an example:

We’ve now been told that we’re no longer required to hold those meetings, but we do so if we find it necessary…For example, when it comes to the home care service, we’ve usually contacted [the person with the right competency] directly—the one in the [consensus group] who’s among those that travel around cleaning people’s homes and who are part of a separate unit. This is something none of the SMEs are very competent in, therefore we’ve made direct contact with her. We’ve had a couple of meetings with her, needs have been explained…[and] she has given us some advice on what we should do. This way, we have used the [consensus] group. Beyond that, it has probably been a while since we’ve used anyone else [from the consensus group], and that’s because we have very competent people, with extensive professional knowledge [among the SMEs], so there hasn’t really been any need [for contacting others]. (Quote 7, R5, leading SME)

R5 describes that, after the breakdown, she and her team of SMEs have changed their practice of how consensus group members are involved in project-related issues. Instead of engaging the consensus group as a whole, as in pre-breakdown group meetings, selected members of the group are now involved whenever issues emerge (in this case, home services) that the development team does have sufficient competencies to settle internally (“This is something none of the SMEs are very competent in.”). As such, the decision about who to involve as issues emerge is based on the consensus group members’ competence, and contact is established directly (“therefore we’ve made direct contact with her”).

R5 also expresses that, given the competencies and level of relevant knowledge among the SMEs that she works with in the project, the need for involving externals has been limited (“it has probably been a while since we’ve used anyone else [from the consensus group], and that’s because we have very competent people, with extensive professional knowledge”). Similar opinions are also expressed in quote 3.

Shifts in involvement practices—from plenary whole-group meetings held at regular intervals to “on-demand” involvement of consensus group members or other project externals—were also reported by other respondents.

#### Discussion partners

Another central feature of the leading SMEs’ collegial involvement practices after the breakdown was the tendency to consult one or a limited set of specific, trusted colleagues (typically SMEs) regarding emerging project-related issues. One leading SME expressed:

It is important to have a discussion partner, simply because you’re a little alone. It’s very nice to have a colleague, not having to stand alone in all the decisions that need to be made…Now, I’ve finally got a pharmacist colleague. (Quote 8, R3, leading SME)

According to the leading SME (R3), a key value associated with having a discussion partner, or a trusted colleague to discuss project-related issues with, is to avoid having to make decisions on issues single-handedly without including others. Having such a partner is regarded as a way of sharing the burden of making decisions.

Describing the nature of the “partnership” between herself and the leading SME who stated the above quote, R4 gave the following account:

[she] presents to me [issues] she thinks have raised some controversy within [the project organization], or [issues] that are a bit difficult. And then we discuss back and forth, and she might get that confirmation from me—when I understand what she’s talking about—that I also agree [with her]. And that’s the way it should be. So, I don’t feel that I really contribute anything new, but maybe I can underscore the decisions she’s making and, in a way, confirm the conclusion she has reached. (Quote 9, R4, colleague in Client organization 2)

R4 describes that the leading SME typically consults her about project-related issues that may be controversial or challenging to make appropriate decisions on. In the quote, R4 suggests that it is through the discussion with the leading SME—likely referring to possibilities for asking follow-questions and further clarification on various aspects—that she develops a sufficient basis for advising the leading SME. R4 sees her role as a discussion partner to be one of helping confirm (or reject) the leading SME’s tentative conclusions based on the information presented, rather than being someone who presents new information of relevance, or radically alternative solutions to the problem presented. From the perspective of R4, then, the discussion with the leading SME is a means for reaching important conclusions in the development of the new EHR system.

In cases where discussion partnerships had been established as a collegial involvement practice, we learned that there were often relationships between the partners prior to the project. Describing her relationship with the leading SME, R4 explained:

We know each other very well. We have known each other since 2002, when we started studying together. So, we have a friendship in addition to a collegial relationship. (Quote 10, R4, colleague in Client organization 2)

R4 does not only have a professional relationship with the leading SME she is collaborating with. The two also have a relationship on a personal level, having been friends for almost 20 years.

As part of the interviews, we also learned about the physical and social contexts under which project-related discussions between a leading SME and their partner took place:

We sit in an open landscape at work on the municipality's premises. We sit right next to each other, and we have a small closet between us, like a dresser-thing, and then it becomes like a "talk over the closet,” or we go to a small quiet room—a meeting room—and talk about it there. (Quote 11, R4, colleague in Client organization 2)

In contrast to the scheduled consensus group meetings, discussions between the leading SMEs and their trusted partner(s) typically occurred spontaneously and often as a partial result of the two parties working within close physical proximity to each other.

As opposed to the “on-demand” involvement described in 5.3.1, the professional partnership between leading SMEs and selected SMEs represents a more stable relation, also characterized by more frequent interaction.

## Summary of the main results

In summary, the interviewees described the declining use of the consensus groups as being related to:

- *Time pressure*: The perceived limited return on time invested in meetings with the consensus group caused the leading SMEs to reduce the frequency of meetings. Too infrequent meetings did not provide timely answers needed by leading SMEs to fulfill project responsibilities and deadlines.
- *Inefficient use of colleagues´ time*: The leading SMEs considered the consensus group meetings to be an inefficient use of colleagues’ time, since the issues being discussed typically concerned only a small subset of the group.
- *Communication and understanding*: The lack of shared terminology and concrete design artifacts aiding the communication between “insiders” and “outsiders” of the project organization limited the exchange of information to a level that was no longer appropriate for the development project.

Regarding post-breakdown collegial involvement, the interviewed leading SMEs described the following two emerging practices:

- *“On-demand” involvement*: Several leading SMEs opted for an ad hoc, competence-oriented involvement practice. This practice involved consulting relevant individual consensus group members whenever specific project-external competencies were required.
- *Discussion partners*: Another reported practice was to involve, on a longer-term basis, one, or a small group of, specific SMEs or other colleagues as consultants in project-related decisions. Typically, the discussion partner was a colleague the leading SME was well acquainted with prior to the project.

**Figure 5.**
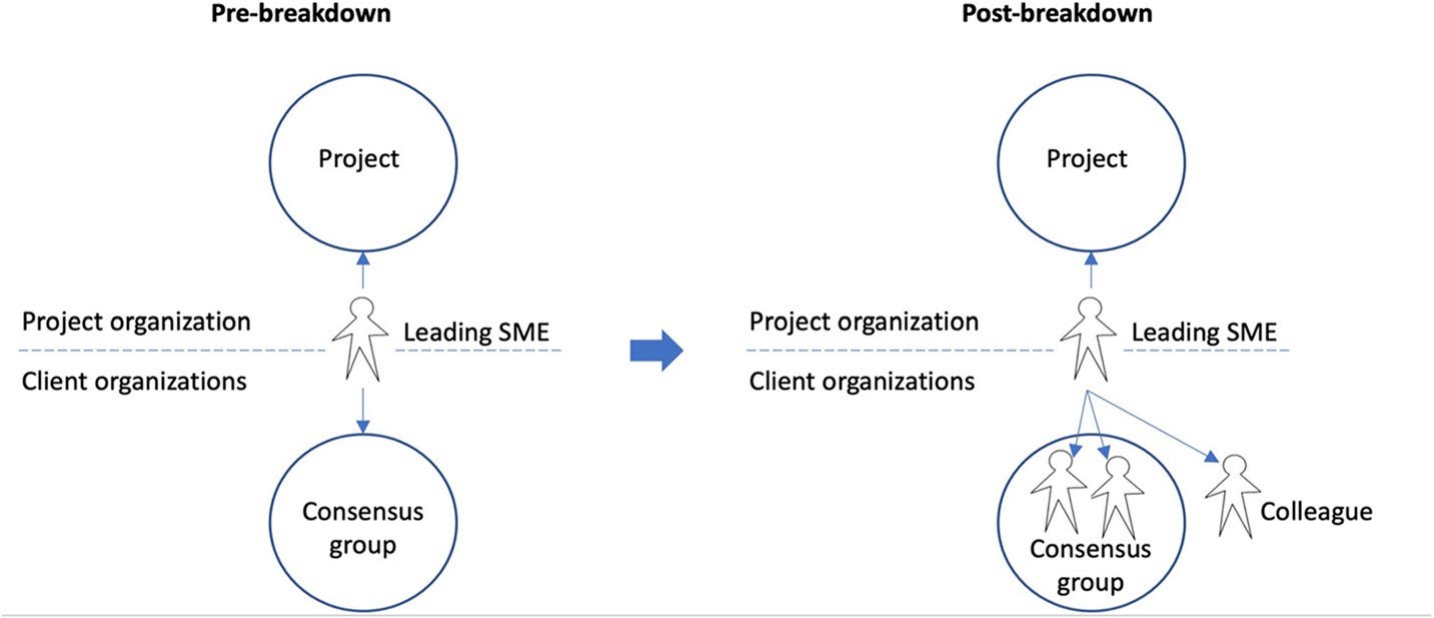
The SMEs’ collegial involvement practices pre-breakdown (left) and post-breakdown (right).

Figure 5 shows a comparison of the SMEs’ collegial involvement practices pre-breakdown and post-breakdown, which involved a shift from relating to the consensus group as a group, to relating to its individual members or other collogues on demand.

## Discussion

Having described central features of the breakdown as perceived by respondents, as well as involvement practices in the aftermath, an interesting question emerges: How can the features and the practices be explained as resulting from the project’s structural arrangements (see Sect. 3)? In the following sections, we first account for potential links between the structural arrangements and the findings. Next, we discuss what we see as the main implications of this study with respect to the practice of involving stakeholder representatives in large-scale projects.

### The breakdown in view of the project’s structural arrangements

#### Time pressure

The leading SMEs considered the consensus group meetings incapable of effectively informing decisions concerning the configuration of the new EHR system in a timely manner. Considering the leading SMEs’ perceived time pressure in light of the project’s structural arrangements, one central problem appears to be that the project timeline and the frequent deadlines that the leading SMEs were required to meet did not match the relatively infrequent meeting schedule of the consensus groups. This situation created an asynchrony between the time-constrained needs of the project and the leading SMEs, on the one hand, and what the consensus group could deliver on time, on the other. The reported tendency among the leading SMEs to decrease the frequency of the consensus group meeting, as a consequence of perceived limited return on time invested, thus led to further asynchrony.

While the consensus groups were established in the preparation phase as advisory units, the issues described above suggest that the consensus groups were not suited to follow the pace of the project after its initiation. They were initiated by the regional public health authority prior to project kick-off and were not prepared for a development process with weekly deliverables.

***Relevant structural arrangements***: The pace of the project, as given by the project timeline with its regular deliverables.

#### Inefficient use of colleagues’ time

Several respondents considered the consensus group meetings to represent inefficient use of colleagues’ time, since the agenda of the meetings typically concerned only a limited set of group members, that is, health care workers with competencies relevant for making near-future deadlines. Taking into account the project’s structural elements, the complexity of the solution and the multiple competencies within the client organizations required to achieve a satisfactory end result appear to have played a central role. Particularly, when the project moved from the more generic specification phase to the more implementation-specific details of the development phase, the broadly represented competencies of the consensus groups were considered excessive vis-à-vis the immediate information needs of the leading SMEs.

***Relevant structural arrangements***: (1) The transition from one project phase to another changes the information needs of the SMEs. (2) Disproportionate representation of competencies in consensus group meetings, as the leading SMEs’ information needs become more related to particular aspects of the new EHR being developed.

#### Communication and understanding

Another central problem the analysis revealed was the challenges in communication and understanding between the (leading) SMEs and the consensus group members. These challenges indicate that the conceptual gap between project “insiders” and project “outsiders” was not sufficiently closed. In view of the project’s structural arrangement, this conceptual gap may potentially be seen as a consequence of the following. First, the leading SMEs acted as primary links between the project and the respective consensus groups (the application analysts in the project organization or the developers in the vendor organization—most based in the US—did not interact directly with the consensus group). As facilitators of the consensus group meetings, the leading SMEs implicitly also became “filters”, as the information they offered represented *their* perspectives and understandings of the development work taking place within the project. As meeting facilitators, the leading SMEs also had a significant impact on the meeting agenda—that is, what aspects of the project work should be given priority.

Second, the pace of the project, and specifically the asynchrony between the project’s continuous information needs and the consensus group meetings, potentially further widened the conceptual gap between project insiders and outsiders. The relatively low frequency of the consensus group meetings gave the members of the consensus groups only a peripheral understanding of the development work, making it challenging to comprehend the rationale behind design proposals emerging from—and design decisions taken within—the project.

***Relevant structural arrangements***: (1) The leading SMEs as the primary “touch-points” or links between the project and the consensus groups. (2) The pace of the project and the resulting asynchrony between the project’s continuous information needs and the consensus group meetings.

#### “On-demand” involvement

While the project’s structural arrangement might help explain the declining influence of the consensus groups (cf. Sections 6.1.1–6.1.3), structural elements may also be seen as playing a role in the collegial practices that to a large degree replaced the consensus groups after the breakdown. When it comes to the practice of “on-demand” involvement, the project’s timeline, with its phases and regular deliverables, can also be seen as a factor leading to more time-efficient collegial involvement practices. At the same time, the establishment of informal involvement practices can only occur in organizational contexts that do not impose strict regulation on involvement practices, but that offer employees a certain degree of autonomy to select practices as they see fit.

Another potential factor that may explain the establishment of “on-demand” involvement, and why it in many cases represented a more preferable alternative than the consensus group meetings, relates to the SMEs (leading and regular) experience from working within and having in-depth knowledge of the client organizations. In most cases where external competencies were required to inform a project-related decision, the leading SMEs, or the team of SMEs they interacted with, knew where to find the correct competencies within the client organizations. This in-depth knowledge of the client organization also made “on-demand” involvement practically feasible.

***Relevant structural arrangements***: (1) The pace of the project, as given by the project timeline with its regular deliverables. (2) No strict regulations on involvement practices. (3) Stakeholder representatives with in-depth knowledge of the client organizations.

#### Discussion partners

The establishment of discussion partners was another collegial involvement practice that replaced the consensus group meetings. We consider the structural arrangements affording “on-demand” involvement (Sect. 6.1.4) to also play a relevant role when it comes to the establishment of discussion partners as an involvement practice. Also, the formation of discussion partnerships can to some extent be seen as a consequence of the challenges related to communication and understanding between the leading SMEs and the consensus groups, and the structural arrangement related to this issue (Sect. 6.1.3)—establishing and maintaining a “common ground” with one specific SME or other colleague can be regarded as less of a burden than doing the same with a consensus group representing a wide variety of competencies. Lastly, and particularly in cases where discussion partnerships were formed between a leading SME and another SME, the sharing of physical workspace (i.e., both having their workplace in the locales of the project organization) and physical proximity appears to have played a factor in the establishment of the practice as it presented the opportunity for ad hoc, informal interactions.

***Relevant structural arrangements***: Same as for “'on-demand involvement” and “communication and understanding,” in addition to sharing of physical workspace.

### Representative participation in view of structural arrangements

In the above, we discussed various ways the structural arrangements of the EHR implementation project can be considered to have affected the SMEs’ collegial involvement practices. The transition involved moving away from relatively large and infrequent consensus group meetings with group members representing a broad set of competencies to more frequent ad hoc interactions with smaller groups or individuals (i.e., *on-demand interaction* and *conversation partners*).

In the following, we discuss some wider implications of our findings when it comes to using representative participation as a strategy to deal with user participation in large-scale IT projects. Specifically, we draw attention to how a project’s structural arrangements and their effects on collegial involvement practices implicitly shape the role of stakeholder representatives, defining to some extent *who* and *what* stakeholder representatives in a project represent.

In his seminal book, *Images of Organization*, Morgan (1986) introduced the use of metaphors to understand and deal with organizational problems. One of the eight organizational metaphors suggested by Morgan was *political systems* (ibid., chapter 6). The essence of the Morgan’s political systems metaphor on organizations is the acknowledgment of conflicting interests that need to be negotiated into a mutually acceptable agreement. To facilitate further discussion, we use a political system, that is, the organizational structure of democracies, as the basis for two analogies (presented in Sects. 6.2.1 and 6.2.2, respectively). The political systems metaphor, however, is applied here for a somewhat different purpose than drawing attention towards negotiation and agreement. Instead, we use the metaphor to highlight two different perspectives on the role of the SMEs in the project and who, or what, they represent.

In a democracy, the supreme arena for political debate and decision-making is the parliament. Parliament is an assembly of representatives of various political interests. The representatives are elected through parliamentary referenda; that is, citizens (the electorate) vote for professional politicians to represent their interests in parliament. A group of voters in a specified area who elect a representative is referred to as a constituency. It is the parliament that determines the composition of the government. Parliament also imposes control of the government and the ministries responsible for implementing government policy.

Modern democracies are far more complex than the structure described above suggests, but for analogical purposes, a simplified account is sufficient. As suggested by Morgan (1986) organizational metaphors are not intended to be exhaustive, but rather means to guide our understanding of organizations and their problems and open new possibilities.

Morgan (ibid., p. 157) lists six “varieties of political rule” for his organization-as-political-system metaphor: Autocracy, Bureaucracy, Technocracy, Codetermination, Representative Democracy, and Direct Democracy. Of these, Direct Democracy corresponds to how we have described participation in early PD projects. For the current case, we have found Representative Democracy and Technocracy to be useful “varieties of political rule” for reflecting on the ambiguous role of the SMEs.

#### Analogy I: SMEs as political representatives

In our first analogy, *SMEs as political representatives*, the central structural elements of the case can be translated into elements found in the organizational structure of representative democracies, as described in Table 1. We can compare the EHR implementation project to that of a democratic state—both requiring governing and decision-making. Similar to the way a parliament acts as the supreme decision-making body of a democratic state, we can think of the project organization as having a similar function in the project (i.e., making decisions concerning the implementation of the new EHR system). Following the logic of the analogy, then, the healthcare professionals in the region would correspond to the electorate (i.e., those entitled to vote in an election), the consensus groups the constituencies, and the SMEs could be seen as the constituency’s representatives in the project organization (parliament). The breakdown, then, is comparable to a loss of public debate (e.g., constituency meetings) between a representative and their constituency. The post-breakdown involvement practices are, in many ways, analogous to a representative acting as if given a *free mandate*, that is, given the authority to act as they see fit (as opposed to an imperative mandate where the holder is required to reflect the will of those they represent).

**Table 1.** Mapping between structural elements in the case and organizational structure of democracy (with SME as political representative).

Case		Representative democracy
EHR project	➔	Democratic state
Project organization	➔	Parliament
Healthcare professionals	➔	Electorate
Consensus group	➔	Constituency
Consensus group meetings	➔	Public constituency meetings
SME	➔	Political representative

#### Analogy II: SMEs as technocrats

However, using an alternative analogy, the SMEs can also be thought of as *technocrats*—technical experts (in this case, experts in medical specialties), especially, those exercising managerial authority (Merriam-Webster, 2022). In such an analogy, the key structural elements in the case can be considered somewhat differently (Table 2). The project organization is analogous to the government, the healthcare professionals the citizens, and the consensus groups advisory units. Rather than seeing the SMEs as representatives of a consensus group (a “constituency”), as in the previous analogy, the SMEs could be regarded as being part of the project organization’s executive bodies responsible for different modules of the EHR system and ensuring that the project plan is followed. According to such a view, then, the SMEs are analogous to politically independent secretaries to the ministries (similar to bureaucrats/technocrats) responsible for implementing government policy, and not bound to the same democratic “rules” as representatives (e.g., participating in public debates). Such an analogy also reflects that the SMEs were not elected by the consensus groups or through a democratic process but appointed to the role (typically by a clinical manager) in light of their medical expertise and experience.

**Table 2.** Mapping between structural elements in the case and organizational structure of democracy (with SME as technocrat).

Case		Technocracy
EHR project	➔	Democratic state
Project organization	➔	Government
Healthcare professionals	➔	Citizens (people affected by the decision)
Consensus group	➔	Advisory unit
Consensus group meetings	➔	Advisory unit meeting
SME	➔	Technocrat

#### Implications

As with all analogies, similarities coexist with significant differences. For example, as pointed out earlier, Analogy I does not reflect that the SMEs were typically appointed, as opposed to democratically elected for the role. Similarly, Analogy II does not reflect the SMEs’ professional relationships with members of the consensus group as co-workers in the client organizations. Regardless, the analogies call attention to how so-called “stakeholder representative” roles in participatory and user-centered projects are formed, to a large extent, not only by the intentions of those inhabiting such roles, but to a great degree by the context in which representative participation takes place. Thus, while the original intention behind the consensus group meetings might have been along the lines of the “SMEs as political representatives” analogy, in which the meetings would ensure participation and deliberation, structural arrangements (e.g., project timeline and deadlines) “forced” the SMEs into a “technocrat” role, that is, acting as implementers of the project plan. Given a deliverable-oriented project plan, the SMEs’ de-prioritization of broad involvement and deliberation can be seen as a consequence, as these aspects were considered obstacles to, rather than means of, realizing the project timeline. The development of a “tribal language” among the SMEs (Quote 4, Sect. 5.2.3) could also reflect the user participants becoming more and more incorporated in the project and, in that way, gradually turning into project representatives rather than remaining user representatives.

Taking related studies on user participation into consideration, there has been relatively little attention paid to how the role of user participants may be affected by a project’s structural elements (i.e., “what shapes participation?” (Bratteteig and Wagner, 2016)) For example, Rasmussen et al. (2011) investigated how users from an intended user population are selected for project participation. While Rasmussen et al. (op.cit.) identified *user advocate* and *system champion* as preferred participant “profiles”— metaphors that to some degree correspond, respectively, with the political representative and technocrat roles of the analogies—their study did not focus on how the participant role may change throughout a project as a result of project conditions. As such, the findings from this investigation complement Rasmussen et al’s. (2011) study.

Similar to how Markus and Mao (2004) argued that activities intended to support either technical development or organizational implementation may inhibit one another, we consider the roles of SME in the two analogies to be potentially contradictory as they imply different priorities. As a political representative, representing “the will” of health professionals (i.e., the user population) comes first. As technocrat (or system champion (Markus and Mao, 2004)), however, the priority is to ensure the progression in the development of the system, while relying, to a greater extent, on personal expertise and experience in related questions.

While answering the question of how to scale up user participation in IT projects is beyond the scope of this paper, it is interesting to note that some emerging issues appear to resemble those associated with democratic governing. To enable empowerment, both require a design or structural arrangements that enable broad involvement, so that representatives can “touch base” with the represented. Concerning large-scale PD projects, Iversen and Dindler (2014) argued that the failure to inform the general user population (i.e., non-participants) about the ongoing PD work risks that they develop negative attitudes towards the project. Drawing on their project experiences, the authors suggested that regular dissemination activities that allow the general user population to see and interact with prototypes emerging from the PD work, while also opening for general critique on the outcomes and the design process, is one way of keeping non-participant “in the loop”. Dissemination activities such as the above, but also the consensus group meetings described earlier, correspond in many ways to what Bødker et al. (2017) describe as the “backstage” design activities—work that goes beyond traditional PD methods and techniques, but which have been identified as important for obtaining the appropriate conditions for PD, particularly in large-scale projects (Iversen and Dindler, 2014; Bødker et al., 2017).

Although representative participation can be seen as one central piece in making large-scale participation in IT projects feasible, this form of participation, and particularly associated backstage activities, nevertheless needs to be planned, to reduce the risk of becoming exclusionary – a “flaw” some political scientists (Dahl, 2008; Landemore, 2017) associate with representative democracy.

## Methodological considerations

Before concluding, we find it appropriate to briefly point out some methodological aspects that should be taken into account when considering the findings from the current study. First, the empirical data was collected by interviewing respondents who were involved directly (as SMEs) or indirectly (through SMEs) in the development work of one specific EHR module. As such, we do not know the extent to which the respondents’ viewpoints also represent the perspectives of SMEs participating in the development of other EHR modules in the project or their non-project affiliated colleagues. Moreover, the reported findings also need to be considered in view of the timing of the interviews. We do not know the degree to which the interviewed SMEs' perspectives and involvement practices may have changed in the aftermath of the study. Thus, the findings represent a “snapshot” in the project’s timeline.

Second, as the initial coding was conducted only by the first author there is a risk of interpretation bias (i.e., the coder wittingly or unwittingly validating themes that were expected or failing to see other possible interpretations of a given transcript excerpt). However, having conducted the interviews in addition to observing several project meetings, the first author was also the author possessing most relevant contextual knowledge pertaining to the case and thus more likely to deduce plausible meanings from respondents’ statements.

Third, while the results presented in Sect. 5—and the subsequent discussion in Sect. 6—draw attention to structural arrangements in the projects and how these may have affected the SMEs’ collegial involvement practices, it should be emphasized that the described arrangements should not be interpreted as an attempt to chart all structural arrangements that may affect the role and practice of participants in a project. The arrangements we have drawn attention to only reflect what could be inferred from the interview transcripts and do not rule out the possibility that other respondents may have called attention to other elements. Since several of the structural arrangements we described are generic in the sense that the same arrangement can be found in most large-scale IT projects (e.g., project timelines with deadlines, user groups with varying needs, participants acting as the primary “touch-points” between the client and the vendor organization), the generalizability of the findings is strengthened.

Four, although we identified issues that from PD’s emancipatory perspective may question the success of the project we have studied, we need to remember that the project was never intended to follow PD principles. Hence, passing a normative “judgment” on the project is not meaningful. Instead, we consider the findings to draw attention to challenges related to large-scale participation, for which there are currently no textbook solutions to be found in PD literature, and which we hope can inspire future investigation.

## Summary and conclusion

This paper has focused on representative participation in large-scale IT projects, using an ongoing health IT project intending to implement a common EHR system in Central Norway as a case study. In their communication to the public, the project is ambiguous as to the role of the participating health professionals (SMEs). Drawing on empirical data from the case study of an EHR implementation project, we investigated the SMEs’ collegial involvement practices and how these changed throughout the project due to various perceived challenges. This change involved a transition from attempts at broad involvement by relatively large advisory units through relatively infrequent meetings to more ad hoc, competence-based involvement of individual colleagues. Drawing on the findings, we discussed how the breakdown in the interaction and the post-breakdown involvement practices can be seen as features pertaining to the structural arrangements relative to the project.

In terms of representative participation in large-scale IT projects, the following key lessons can be drawn from the present study:

- **Project structure affects the level of participation:** The collegial involvement practices of users involved in a project’s participatory activities are likely to be affected by the project’s structural arrangements and how it is organized. Thus, possibilities for broad involvement and deliberation are conditioned and constrained by the arrangements.
- **Project structure affects role:** A project’s structural arrangements implicitly direct who and what participants in a project represent. Structural constraints (e.g., project pace and frequent deliverables) can effectively turn user representatives into representatives of their own interests and perspectives, or “technocrats” of the project, or a mix of the above. It is therefore important that large-scale participatory projects take into consideration the role of the participating users and who, or what, they represent. Similarly, it is essential that it is made explicit for the participating users what their role entails.
- **Representative participation requires rethinking project structure:** Representative participation in large-scale IT projects, as a means to deal with the problems associated with the direct involvement of large user groups, raises issues that in many ways are comparable with those associated with representative democracy. Both require structural arrangements, that reduce the risk of representative participation becoming exclusionary. Interactions between representatives and those represented hence need to be reflected in the design of large-scale IT projects.

We hope that the current study motivates further research on the topic of representative participation in large-scale IT projects.

## Footnotes

1 *“fagmiljø” (Norwegian).*

2 *“fageksperter” (Norwegian).*
